# Supplementary material for: Metabolic retroconversion of trimethylamine N-oxide and the gut microbiota
Source: Microbiome. 2018 Apr 20;6:73. doi: 10.1186/s40168-018-0461-0 (PMC5909246; doi:10.1186/s40168-018-0461-0)
Supplement: Supplementary file 1 — Figure S1. Effect of TMAO on mixed faecal microbial population in vitro. Enumeration of bacteria in fermentation vessels by FISH analysis. Red lines, TMAO-containing systems; blue lines, negative controls. Data are shown as mean + SD (n = 3). Eub338, total bacteria; Bac303, Bacteroidales; Ato291, “Atopobium cluster”; Bif164, Bifidobacterium spp.; Ent, Enterobacteriaceae; Bet42a, Betaproteobacteria; Prop853, Veillonellaceae; Lab158, lactic acid bacteria (Lactobacillales); Fpra655, Faecalibacterium prausnitzii and Subdoligranulum spp.; Erec482, Lachnospiraceae; Clit135, Clostridium cluster XI; Rbro730/Rfla729, Ruminococcaceae; Chis150, Clostridium clusters I and II. Detection limit of the method was 4.95 log10 (number of bacteria/mL sample). *Statistically significantly different (adjusted P < 0.05) from the control at the same time point. Table S1. Oligonucleotide probes used in this study. Table S2. NMR-based analysis of metabolites in growth medium of pure cultures after 24-h incubation (anaerobic) in the presence and absence of TMAO. Table S3. FISH data for the batch culture samples, with data presented as log10 (number of bacteria/mL sample). Table S4. NMR data for the batch culture samples, with concentrations of metabolites given in mM. Table S5. Spearman correlation (corrected for ties) and adjusted P values (Benjamini–Hochberg) for FISH and metabolite data for batch culture samples. (DOCX 227 kb) [file 40168_2018_461_MOESM1_ESM.docx]

**SUPPLEMENTARY MATERIAL**


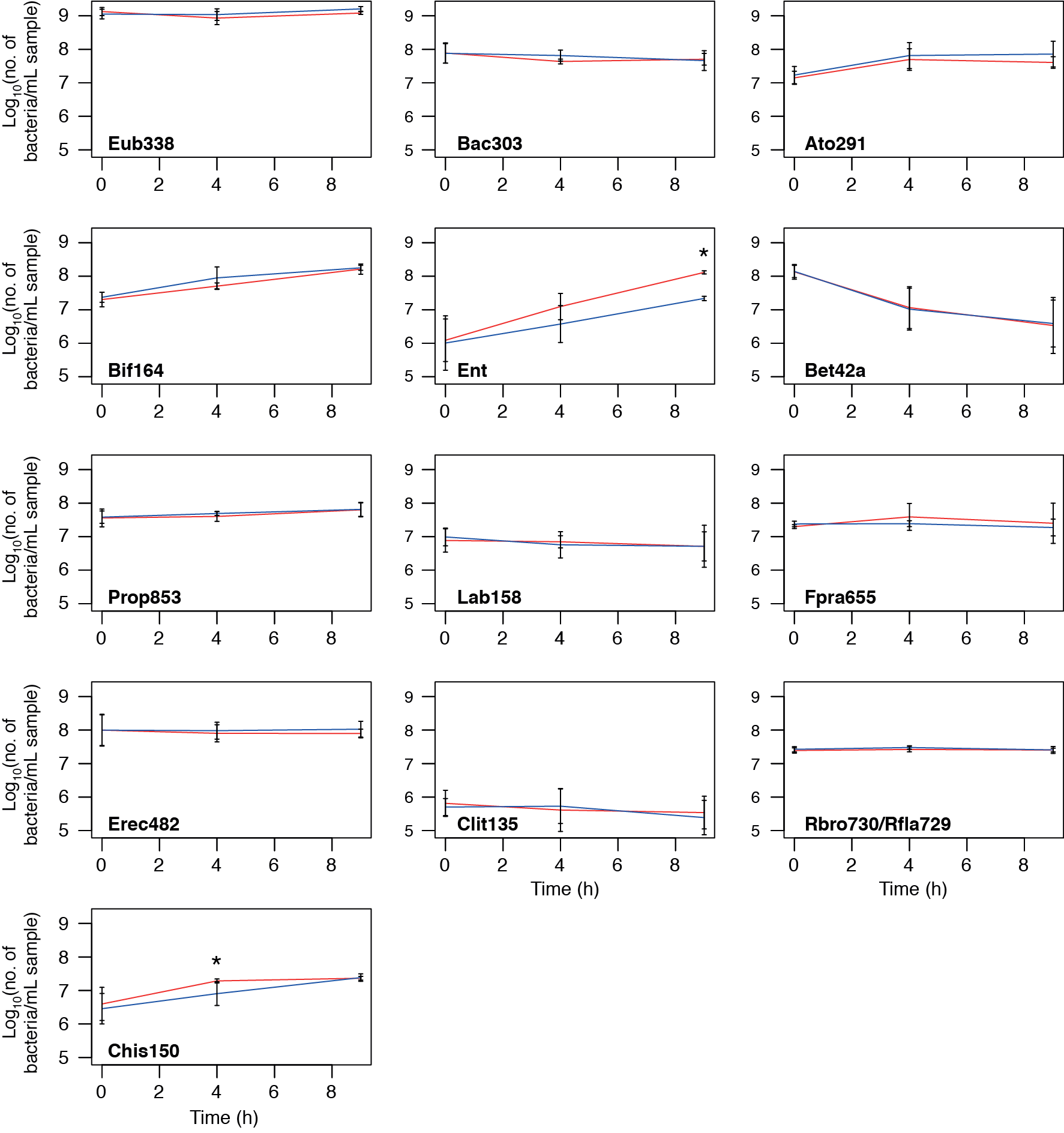


**Supplementary Figure 1.** Effect of TMAO on mixed faecal microbial population *in vitro*. Enumeration of bacteria in fermentation vessels by FISH analysis. Red lines, TMAO-containing systems; blue lines, negative controls. Data are shown as mean + sd (*n* = 3). Eub338, total bacteria; Bac303, *Bacteroidales*; Ato291, ‘*Atopobium* cluster’; Bif164, *Bifidobacterium* spp.; Ent, *Enterobacteriaceae*; Bet42a, *Betaproteobacteria*; Prop853, *Veillonellaceae*; Lab158, lactic acid bacteria (*Lactobacillales*); Fpra655, *Faecalibacterium prausnitzii* and *Subdoligranulum* spp.; Erec482, *Lachnospiraceae*; Clit135, *Clostridium* cluster XI; Rbro730/Rfla729, *Ruminococcaceae*; Chis150, *Clostridium* clusters I and II. Detection limit of the method was 4.95 log_10_(number of bacteria/mL sample). *, Statistically significantly different (adjusted *P* < 0.05) from the control at the same time point.

**Supplementary Table 1.** Oligonucleotide probes used in this study

| **Probe name** | **Detects** | **Probe accession no.*** | **Hybridization pre-treatment**† | **Formamide (%) in hybridization buffer** | **Temperature (°C)** | | **Reference(s)** |
| --- | --- | --- | --- | --- | --- | --- | --- |
|  |  |  |  |  | **Hybridization** | **Washing** |  |
| Ato291 | *Cryptobacterium curtum*, *Gordonibacter* *pamelaeae*, *Paraeggerthella hongkongensis*, all *Eggerthella*, *Collinsella*, *Olsenella* and *Atopobium* species; ‘*Enorma*’ and *Senegalemassilia* spp. (most members of the class *Coriobacteriia*; phylum *Actinobacteria*) | pB-00943 | Lysozyme | 0 | 50 | 50 | Harmsen *et al.* (2000); Thorasin *et al.* (2015) |
| Bac303 | Most members of the genus *Bacteroides*, some *Parabacteroides* and *Prevotella* species, *Paraprevotella*, *Xylanibacter*, *Barnesiella* species and *Odoribacter* *splanchnicus* (order *Bacteroidales*; phylum *Bacteroidetes*) | pB-00031 | None | 0 | 46 | 48 | Manz *et al.* (1996); Hoyles and McCartney (2009) |
| Bet42a | *Betaproteobacteria* (class *Betaproteobacteria*; phylum *Proteobacteria*) | pB-00034 | None | 35 | 46 | 48 | Manz *et al.* (1992) |
| Bif164 | Most *Bifidobacterium* species and *Parascardovia denticolens* (family *Bifidobacteriaceae*; phylum *Actinobacteria*) | pB-00037 | Lysozyme | 0 | 50 | 50 | Langendijk et al. (1995) |
| Chis150 | Most members of *Clostridium* cluster I, all members of *Clostridium* cluster II (family *Clostridiaceae*; phylum *Firmicutes*) | pB-00962 | None | 0 | 50 | 50 | Franks *et al.* (1998) |
| Clit135 | Nine members of [*Clostridium*] cluster XI, including [*Clostridium*] *difficile* (family *Peptostreptococcaceae*; phylum *Firmicutes*) | pB-00961 | None | 0 | 50 | 50 | Franks *et al.* (1998) |
| Ent | *Enterobacteriaceae* except *Proteus* spp. (order *Gammaproteobacteria*; phylum *Proteobacteria*) | pB-00351 | None | 30 | 46 | 48 | Kempf *et al.* (2000) |
| Erec482 | Most members of *Clostridium* cluster XIVa (family *Lachnospiraceae*; phylum *Firmicutes*) | pB-00963 | None | 0 | 50 | 50 | Franks *et al.* (1998) |
| Eub338/  Eub338II/  Eub338III‡ | *Bacteria* | pB-00159/  pB-00160/  pB-00161 | None | 35 | 46 | 48 | Daims *et al.* (1999) |
| Fpra655 | *Faecalibacterium prausnitzii* and *Subdoligranulum* spp. (*Clostridium* cluster IV; family *Ruminococcaceae*; phylum *Firmicutes*) | pB-00734 | None | 0 | 58 | 58 | Hold *et al.* (2003) |
| Lab158 | All *Oenococcus*, *Vagococcus*, *Melissococcus*, *Tetragenococcus*, *Enterococcus*, *Catellicoccus*, *Paralactobacillus*, *Pediococcus* and *Lactococcus* species, most *Lactobacillus*, *Weissella* and *Leuconostoc* species (order *Lactobacillales*; phylum *Firmicutes*) | pB-03928 | Lysozyme | 0 | 50 | 50 | Harmsen *et al.* (1999) |
| Prop853 | Most members of *Clostridium* cluster IX (family *Veillonellaceae*; phylum *Firmicutes*) | pB-03930 | None | 0 | 50 | 50 | Walker *et al.* (2005) |
| Rbro730/  Rfla729‡ | *Anaerotruncus colihominis*, *Ruminococcus bromii*, *Ruminococcus* *flavefaciens*, *Ruminococcus albus*, *Desulfotomaculum alcoholivorax* (Clostridium cluster IV; family *Ruminococcaceae*; phylum *Firmicutes*) | pB-00558/  pB-00557 | Lysozyme | 20 | 50 | 50 | Harmsen *et al.* (2002) |

*According to probeBase (Loy *et al.*, 2007).

†As described by Martín-Peláez *et al.* (2008).

‡These probes were used together in equimolar concentrations (each at 50 ng/μL).

**Supplementary** **Table 2.** NMR-based analysis of metabolites in growth medium of pure cultures after 24-h incubation (anaerobic) in the presence and absence of TMAO

| **Species and isolate*** | **Family, phylum** | **TMA (mM)** | | **DMA (mM)** | | **Acetate (mM)** | | **Ethanol** | | **Lactate** | |
| --- | --- | --- | --- | --- | --- | --- | --- | --- | --- | --- | --- |
|  |  | **+TMAO** | **-TMAO** | **+TMAO** | **-TMAO** | **+TMAO** | **-TMAO** | **+TMAO** | **-TMAO** | **+TMAO** | **-TMAO** |
| ***Escherichia coli* L6-FAA1** | *Enterobacteriaceae*, *Proteobacteria* | 53.94 | 0.03 | 0.03 | 0.05 | 28.17 | 0.70 | 7.64 | 8.20 | 0.19 | 5.10 |
| ***Escherichia coli* L24-FAA5** | *Enterobacteriaceae*, *Proteobacteria* | 53.94 | 0.04 | 0.07 | 0.05 | 27.67 | 0.56 | 7.14 | 4.00 | 2.40 | 6.00 |
| ***Escherichia coli* L9-MRS1** | *Enterobacteriaceae*, *Proteobacteria* | 52.94 | 0.02 | 0.08 | 0.04 | 27.17 | 0.60 | 9.14 | 10.20 | 0.00 | 4.70 |
| ***Escherichia coli* L19-FAA2** | *Enterobacteriaceae*, *Proteobacteria* | 51.94 | 0.03 | 0.03 | 0.05 | 24.17 | 0.60 | 7.64 | 9.80 | 0.02 | 4.80 |
| ***Hafnia paralvei* L15-FAA9** | *Enterobacteriaceae*, *Proteobacteria* | 45.44 | 0.03 | 0.08 | 0.07 | 23.97 | 5.20 | 6.44 | 14.60 | 0.80 | 2.50 |
| ***Escherichia coli* L13-FAA2** | *Enterobacteriaceae*, *Proteobacteria* | 44.94 | 0.03 | 0.00 | 0.04 | 23.47 | 0.60 | 8.64 | 10.00 | 0.00 | 4.50 |
| ***Escherichia coli* L16-FAA5** | *Enterobacteriaceae*, *Proteobacteria* | 44.94 | 0.03 | 0.06 | 0.05 | 23.17 | 0.60 | 9.64 | 9.30 | 0.06 | 4.80 |
| ***Escherichia coli* L20-FAA3** | *Enterobacteriaceae*, *Proteobacteria* | 43.94 | 0.04 | 0.07 | 0.07 | 24.17 | 0.70 | 12.64 | 9.00 | 0.15 | 6.50 |
| ***Escherichia coli* L1-FAA5** | *Enterobacteriaceae*, *Proteobacteria* | 42.94 | 0.03 | 0.03 | 0.05 | 23.67 | 0.60 | 10.64 | 9.00 | 0.03 | 5.20 |
| ***Citrobacter koseri* L8-FAA3** | *Enterobacteriaceae*, *Proteobacteria* | 42.24 | 0.02 | 0.35 | 0.04 | 21.17 | 0.74 | 10.64 | 11.00 | 5.30 | 5.00 |
| ***Escherichia coli* L5-FAA2** | *Enterobacteriaceae*, *Proteobacteria* | 41.94 | 0.02 | 0.07 | 0.03 | 23.17 | 0.60 | 10.64 | 9.70 | 0.32 | 4.10 |
| ***Citrobacter gillenii* L26-FAA1** | *Enterobacteriaceae*, *Proteobacteria* | 37.94 | 0.03 | 0.13 | 0.06 | 18.77 | 0.60 | 12.64 | 11.00 | 4.60 | 5.40 |
| ***Escherichia coli* D3(8)** | *Enterobacteriaceae*, *Proteobacteria* | 32.64 | 0.04 | 0.03 | 0.08 | 16.77 | 6.80 | 6.14 | 6.90 | 0.00 | 8.70 |
| ***Escherichia coli* D2(8)** | *Enterobacteriaceae*, *Proteobacteria* | 31.84 | 0.03 | 0.02 | 0.06 | 16.47 | 8.20 | 6.74 | 7.00 | 0.00 | 8.50 |
| ***Escherichia coli* D2(2)** | *Enterobacteriaceae*, *Proteobacteria* | 31.54 | 0.03 | 0.01 | 0.06 | 16.27 | 8.00 | 6.34 | 6.80 | 0.00 | 9.50 |
| ***Escherichia coli* D1(2)** | *Enterobacteriaceae*, *Proteobacteria* | 30.34 | 0.03 | 0.00 | 0.07 | 16.17 | 8.60 | 5.44 | 8.00 | 0.00 | 9.40 |
| ***Escherichia coli* D4(15)** | *Enterobacteriaceae*, *Proteobacteria* | 29.94 | 0.03 | 0.03 | 0.06 | 14.67 | 6.75 | 6.64 | 6.40 | 0.20 | 7.30 |
| ***Escherichia coli* D2(1)** | *Enterobacteriaceae*, *Proteobacteria* | 24.44 | 0.05 | 0.00 | 0.08 | 11.67 | 8.70 | 4.14 | 7.60 | 0.00 | 9.00 |
| ***Escherichia coli* D5(1)** | *Enterobacteriaceae*, *Proteobacteria* | 22.94 | 0.04 | 0.00 | 0.05 | 12.37 | 6.70 | 5.14 | 6.40 | 0.00 | 7.00 |
| ***Klebsiella pneumoniae* subsp. *pneumoniae* L4-FAA5** | *Enterobacteriaceae*, *Proteobacteria* | 14.94 | 0.02 | 0.41 | 0.05 | 10.17 | 0.52 | 7.14 | 12.00 | 4.40 | 5.90 |
| *Clostridium sporogenes* D1(9) | *Clostridiaceae* (Cluster I), *Firmicutes* | 4.64 | 0.12 | 1.03 | 0.07 | 17.37 | 16.90 | 16.24 | 13.50 | 0.10 | 0.90 |
| [*Clostridium*] *bifermentans* D4(1) | *Peptostreptococcaceae*, *Firmicutes* | 3.72 | 0.00 | 1.08 | 0.00 | 12.15 | 0.00 | 8.81 | 0.00 | 0.03 | 0.00 |
| *Clostridium perfringens* L20-BSM1 | *Clostridiaceae* (Cluster I), *Firmicutes* | 2.19 | 0.03 | 0.23 | 0.06 | 1.97 | 0.81 | 10.64 | 5.30 | 4.80 | 5.90 |
| *Enterococcus gallinarum* D6(5) | *Enterococcaceae*, *Firmicutes* | 1.74 | 0.04 | 0.30 | 0.15 | 0.00 | 0.77 | 1.24 | 0.83 | 29.80 | 2.00 |
| *Bacteroides vulgatus* L9-FAA7 | *Bacteroidaceae*, *Bacteroidetes* | 1.70 | 0.00 | 0.88 | 0.00 | 2.65 | 0.00 | 0.00 | 0.00 | 0.00 | 0.00 |
| *Enterococcus faecalis* D5(2) | *Enterococcaceae*, *Firmicutes* | 1.64 | 0.05 | 0.45 | 0.13 | 0.00 | 0.67 | 0.82 | 1.04 | 27.80 | 1.60 |
| *Parabacteroides johnsonii* L13-FAA10 | *Porphyromonadaceae*, *Bacteroidetes* | 1.42 | 0.00 | 0.65 | 0.00 | 1.51 | 0.00 | 0.00 | 0.00 | 0.24 | 0.00 |
| *Bacteroides fragilis* L6-FAA7 | *Bacteroidaceae*, *Bacteroidetes* | 1.42 | 0.00 | 0.51 | 0.00 | 2.67 | 0.00 | 0.00 | 0.00 | 0.56 | 0.00 |
| *Enterococcus faecalis* D3(1) | *Enterococcaceae*, *Firmicutes* | 1.24 | 0.06 | 0.29 | 0.16 | 0.00 | 0.74 | 1.24 | 1.02 | 28.60 | 2.00 |
| *Bacteroides vulgatus* D1(4) | *Bacteroidaceae*, *Bacteroidetes* | 1.08 | 0.02 | 0.71 | 0.01 | 1.66 | 2.82 | 0.06 | 0.00 | 0.12 | 0.33 |
| *Clostridium paraputrificum* L16-FAA6 | *Clostridiaceae* (Cluster I), *Firmicutes* | 1.04 | 0.03 | 0.33 | 0.07 | 1.37 | 0.63 | 1.04 | 2.50 | 2.00 | 3.60 |
| *Enterococcus faecalis* D2(14) | *Enterococcaceae*, *Firmicutes* | 1.00 | 0.05 | 0.17 | 0.14 | 0.00 | 0.69 | 1.34 | 1.40 | 27.20 | 1.80 |
| [*Clostridium*] *ramosum* L16-FAA1 | *Erysipelotrichaceae*, *Firmicutes* | 0.94 | 0.07 | 0.33 | 0.09 | 1.47 | 0.60 | 1.34 | 1.80 | 4.30 | 5.20 |
| *Streptococcus sanguinis* L4-MRS1 | *Streptococcaceae*, *Firmicutes* | 0.49 | 0.03 | 0.02 | 0.06 | 0.00 | 0.60 | 0.34 | 0.33 | 22.90 | 9.20 |
| *Streptococcus anginosus* D5(12) | *Streptococcaceae*, *Firmicutes* | 0.48 | 0.02 | 0.16 | 0.12 | 0.00 | 0.60 | 0.09 | 0.46 | 29.30 | 2.00 |
| *Streptococcus anginosus* D1(5) | *Streptococcaceae*, *Firmicutes* | 0.44 | 0.03 | 0.11 | 0.14 | 0.00 | 0.64 | 0.06 | 0.38 | 29.10 | 2.20 |
| *Bifidobacterium breve* LCR5 | *Bifidobacteriaceae*, *Actinobacteria* | 0.38 | 0.03 | 0.04 | 0.00 | 32.04 | 26.51 | 2.00 | 0.89 | 12.77 | 0.68 |
| *Staphylococcus hominis* D2(4) | *Staphylococcaceae*, *Firmicutes* | 0.34 | 0.03 | 0.33 | 0.08 | 1.47 | 2.30 | 0.08 | 0.32 | 2.50 | 4.00 |
| *Enterococcus faecium* D6(1) | *Enterococcaceae*, *Firmicutes* | 0.31 | 0.06 | 0.13 | 0.14 | 0.00 | 0.70 | 0.30 | 0.37 | 30.80 | 1.95 |
| *Bifidobacterium infantis* DSM 20088T | *Bifidobacteriaceae*, *Actinobacteria* | 0.27 | 0.02 | 0.04 | 0.00 | 27.56 | 29.81 | 0.34 | 0.21 | 12.01 | 0.94 |
| *Streptococcus vestibularis* L26-MRS7 | *Streptococcaceae*, *Firmicutes* | 0.25 | 0.02 | 0.00 | 0.05 | 0.00 | 0.40 | 0.28 | 0.65 | 29.40 | 2.20 |
| *Bifidobacterium bifidum* LCR11 | *Bifidobacteriaceae*, *Actinobacteria* | 0.24 | 0.02 | 0.02 | 0.01 | 33.14 | 33.91 | 0.50 | 0.49 | 14.13 | 0.91 |
| *Lactobacillus fermentum* L26-MRS5 | *Lactobacillaceae*, *Firmicutes* | 0.22 | 0.02 | 0.16 | 0.03 | 0.47 | 1.50 | 0.19 | 0.80 | 0.70 | 2.70 |
| *Bifidobacterium longum* LCR6 | *Bifidobacteriaceae*, *Actinobacteria* | 0.22 | 0.02 | 0.02 | 0.01 | 23.86 | 23.77 | 0.39 | 0.06 | 10.47 | 0.77 |
| *Bifidobacterium longum* DSM 20219^T^ | *Bifidobacteriaceae*, *Actinobacteria* | 0.20 | 0.02 | 0.02 | 0.00 | 27.92 | 23.19 | 0.58 | 0.28 | 13.51 | 0.83 |
| *Bifidobacterium longum* subsp. *infantis* LCR2 | *Bifidobacteriaceae*, *Actinobacteria* | 0.18 | 0.03 | 0.02 | 0.01 | 25.11 | 0.00 | 0.38 | 0.02 | 11.65 | 0.74 |
| *Streptococcus oralis* L4-MRS5 | *Streptococcaceae*, *Firmicutes* | 0.16 | 0.02 | 0.00 | 0.05 | 0.00 | 0.60 | 1.39 | 0.80 | 24.60 | 5.10 |
| *Streptococcus* sp. D4(3) | *Streptococcaceae*, *Firmicutes* | 0.16 | 0.03 | 0.06 | 0.13 | 0.00 | 0.56 | 0.25 | 0.40 | 27.90 | 1.90 |
| *Streptococcus* *gallolyticus* L25-MRS1 | *Streptococcaceae*, *Firmicutes* | 0.14 | 0.04 | 0.00 | 0.06 | 0.00 | 0.60 | 0.09 | 0.35 | 31.20 | 2.20 |
| *Bifidobacterium pseudocatenulatum* LCR3 | *Bifidobacteriaceae*, *Actinobacteria* | 0.14 | 0.03 | 0.01 | 0.02 | 28.26 | 19.68 | 0.78 | 0.27 | 12.66 | 1.25 |
| *Pseudomonas aeruginosa* L1-FAA6 | *Pseudomonadaceae*, *Proteobacteria* | 0.12 | 0.02 | 0.04 | 0.00 | 0.09 | 0.00 | 0.03 | 0.00 | 0.00 | 0.00 |
| *Lactobacillus rhamnosus* L26-FAA6 | *Lactobacillaceae*, *Firmicutes* | 0.12 | 0.03 | 0.02 | 0.00 | 0.16 | 0.00 | 0.11 | 0.00 | 12.49 | 0.00 |
| *Bifidobacterium animalis* subsp. *lactis* L26-MRS4 | *Bifidobacteriaceae*, *Actinobacteria* | 0.10 | 0.02 | 0.01 | 0.00 | 6.76 | 6.32 | 0.10 | 0.01 | 3.12 | 2.73 |
| *Bifidobacterium longum* subsp. *longum* L19-MRS1 | *Bifidobacteriaceae*, *Actinobacteria* | 0.09 | 0.02 | 0.01 | 0.01 | 14.86 | 12.64 | 0.22 | 0.13 | 6.79 | 3.06 |
| Unknown *Bifidobacterium* LCR4 | *Bifidobacteriaceae*, *Actinobacteria* | 0.07 | 0.02 | 0.01 | 0.00 | 8.51 | 6.84 | 0.06 | 0.00 | 4.10 | 3.75 |
| *Bifidobacterium breve* LCR8 | *Bifidobacteriaceae*, *Actinobacteria* | 0.05 | 0.02 | 0.01 | 0.00 | 13.11 | 6.33 | 0.14 | 0.00 | 6.07 | 3.49 |
| *Bifidobacterium longum* subsp. *longum* L25-MRS8 | *Bifidobacteriaceae*, *Actinobacteria* | 0.05 | 0.02 | 0.02 | 0.01 | 5.87 | 9.40 | 0.09 | 0.05 | 2.63 | 4.68 |
| *Bifidobacterium bifidum* L25-MRS2 | *Bifidobacteriaceae*, *Actinobacteria* | 0.05 | 0.02 | 0.01 | 0.01 | 1.32 | 1.72 | 0.06 | 0.07 | 0.34 | 0.90 |
| *Bifidobacterium dentium* LCR1 | *Bifidobacteriaceae*, *Actinobacteria* | 0.04 | 0.02 | 0.02 | 0.00 | 2.18 | 1.75 | 0.04 | 0.01 | 0.88 | 1.14 |
| [*Clostridium*] *innocuum* D2(9) | *Erysipelotrichaceae*, *Firmicutes* | 0.03 | 0.03 | 0.01 | 0.00 | 0.26 | 0.00 | 0.04 | 0.00 | 0.10 | 0.00 |
| *Bifidobacterium adolescentis* DSM 20083^T^ | *Bifidobacteriaceae*, *Actinobacteria* | 0.02 | 0.02 | 0.01 | 0.00 | 1.85 | 2.31 | 0.02 | 0.01 | 0.92 | 1.57 |
| *Bifidobacterium animalis* subsp. *lactis* LCR26 | *Bifidobacteriaceae*, *Actinobacteria* | 0.02 | 0.02 | 0.01 | 0.00 | 1.26 | 1.75 | 0.01 | 0.06 | 0.39 | 0.79 |
| *Actinomyces odontolyticus* L12-BSM1 | *Actinomycetaceae*, *Actinobacteria* | 0.02 | 0.02 | 0.01 | 0.00 | 0.82 | 0.00 | 0.00 | 0.00 | 0.02 | 0.00 |
| *Fusobacterium ulcerans* L9-FAA5 | *Fusobacteriaceae*, *Fusobacteria* | 0.02 | 0.02 | 0.02 | 0.00 | 0.14 | 1.46 | 0.07 | 0.00 | 0.00 | 0.10 |
| *Bifidobacterium gallicum* DSM 20093^T^ | *Bifidobacteriaceae*, *Actinobacteria* | 0.01 | 0.01 | 0.01 | 0.00 | 0.31 | 0.33 | 0.02 | 0.00 | 0.06 | 0.43 |
| *Actinomyces viscosus* L6-BSM10 | *Actinomycetaceae*, *Actinobacteria* | 0.00 | 0.00 | 0.00 | 0.00 | 0.04 | 0.22 | 0.03 | 0.00 | 0.00 | 0.06 |

*Species and isolates shown in bold produce large quantities of TMA from TMAO.

**Supplementary Table 3.** FISH data for the batch culture samples, with data presented as log_10_(number of bacteria/mL sample)

| **System, time (h)*** | **EUB338** | **Bac303** | **Ato291** | **Bif164** | **Ent** | **Bet42a** | **Prop853** | **Lab158** | **Fpra655** | **Erec482** | **Clit135** | **Rbro/Rfla** | **Chis150** |
| --- | --- | --- | --- | --- | --- | --- | --- | --- | --- | --- | --- | --- | --- |
| A0 | 9.08 | 7.78 | 6.96 | 7.17 | 5.65 | 8.22 | 7.68 | 6.70 | 7.36 | 8.52 | 6.21 | 7.44 | 7.05 |
| A4 | 8.89 | 7.70 | 7.40 | 7.64 | 7.36 | 7.32 | 7.44 | 7.04 | 7.41 | 8.20 | 5.65 | 7.46 | 7.34 |
| A9 | 9.13 | 7.90 | 7.48 | 8.04 | 8.10 | 6.10 | 7.62 | 6.92 | 7.24 | 7.98 | 5.25 | 7.39 | 7.30 |
| B0 | 8.97 | 7.81 | 6.95 | 7.26 | 5.65 | 8.22 | 7.69 | 6.93 | 7.45 | 8.54 | 5.99 | 7.49 | 6.84 |
| B4 | 8.99 | 7.80 | 7.43 | 7.57 | 7.01 | 7.37 | 7.62 | 7.20 | 7.43 | 8.26 | 5.65 | 7.42 | 7.08 |
| B9 | 9.25 | 7.92 | 7.63 | 8.17 | 7.40 | 6.18 | 7.63 | 6.98 | 7.42 | 8.11 | 4.95 | 7.40 | 7.27 |
| C0 | 9.03 | 7.65 | 7.14 | 7.19 | 5.80 | 8.29 | 7.74 | 6.67 | 7.25 | 7.70 | 5.43 | 7.30 | 6.07 |
| C4 | 8.76 | 7.56 | 7.64 | 7.81 | 6.64 | 6.35 | 7.74 | 6.81 | 7.31 | 7.73 | 4.95 | 7.34 | 7.22 |
| C9 | 9.05 | 7.57 | 7.54 | 8.33 | 8.17 | 5.99 | 7.76 | 6.21 | 6.89 | 7.75 | 5.25 | 7.36 | 7.41 |
| D0 | 8.97 | 7.63 | 7.30 | 7.31 | 5.43 | 8.27 | 7.69 | 6.76 | 7.29 | 7.67 | 5.56 | 7.44 | 5.95 |
| D4 | 8.89 | 7.66 | 7.80 | 8.12 | 5.95 | 6.30 | 7.71 | 6.47 | 7.29 | 7.77 | 5.25 | 7.53 | 6.50 |
| D9 | 9.12 | 7.73 | 7.65 | 8.26 | 7.33 | 6.18 | 7.77 | 5.99 | 6.98 | 7.76 | 5.25 | 7.31 | 7.49 |
| E0 | 9.26 | 8.23 | 7.35 | 7.55 | 6.82 | 7.88 | 7.25 | 7.29 | 7.29 | 7.77 | 5.80 | 7.43 | 6.68 |
| E4 | 9.15 | 7.65 | 8.04 | 7.66 | 7.28 | 7.51 | 7.63 | 6.69 | 8.05 | 7.78 | 6.23 | 7.47 | 7.29 |
| E9 | 9.07 | 7.63 | 7.80 | 8.26 | 8.07 | 7.49 | 8.03 | 7.00 | 8.06 | 7.97 | 6.10 | 7.47 | 7.38 |
| F0 | 9.22 | 8.20 | 7.45 | 7.54 | 6.94 | 7.94 | 7.37 | 7.28 | 7.39 | 7.78 | 5.56 | 7.34 | 6.58 |
| F4 | 9.23 | 7.98 | 8.21 | 8.16 | 6.76 | 7.39 | 7.73 | 6.60 | 7.45 | 7.92 | 6.28 | 7.48 | 7.14 |
| F9 | 9.24 | 7.34 | 8.30 | 8.32 | 7.28 | 7.40 | 8.04 | 7.16 | 7.42 | 8.21 | 5.95 | 7.51 | 7.39 |

*A prefix, donor 1 vessel containing TMAO; B prefix, donor 1 vessel without TMAO (negative control); C prefix, donor 2 vessel containing TMAO; D prefix, donor 2 vessel without TMAO (negative control); E prefix, donor 3 vessel containing TMAO; F prefix, donor 3 vessel without TMAO (negative control).

**Supplementary Table 4.** NMR data for the batch culture samples, with concentrations of metabolites given in mM

| **System, time (h)** | **TMA** | **TMAO** | **DMA** | **Ethanol** | **Acetate** | **Lactate** |
| --- | --- | --- | --- | --- | --- | --- |
| A0 | 0.03 | 89.45 | 0.05 | 0.44 | 1.63 | 1.44 |
| A4 | 0.77 | 92.3 | 0.45 | 0.59 | 6.68 | 3.69 |
| A9 | 5.46 | 71.4 | 0.86 | 1.76 | 26.51 | 13.61 |
| B0 | 0.03 | 0.58 | 0.04 | 0.31 | 1.51 | 1.42 |
| B4 | 0.03 | 0.56 | 0.03 | 0.92 | 9.79 | 4.53 |
| B9 | 0.06 | 0.21 | 0.05 | 2.14 | 33.5 | 12 |
| C0 | 0.02 | 80 | 0.04 | 0.4 | 0.89 | 1.44 |
| C4 | 0.16 | 81.1 | 0.19 | 0.55 | 4.61 | 2.96 |
| C9 | 6.33 | 76.83 | 0.4 | 2.07 | 33.7 | 15.51 |
| D0 | 0.03 | 0.62 | 0.05 | 0.31 | 0.97 | 1.5 |
| D4 | 0.03 | 0.55 | 0.03 | 0.69 | 6.41 | 3.74 |
| D9 | 0.06 | 0.2 | 0.04 | 2.01 | 32.4 | 13.94 |
| E0 | 0.03 | 79.6 | 0.05 | 0.41 | 1.76 | 1.4 |
| E4 | 1.61 | 80 | 0.61 | 1.52 | 17.5 | 7.38 |
| E9 | 10.81 | 70.8 | 1.4 | 2.5 | 38.5 | 10.7 |
| F0 | 0.03 | 0.58 | 0.05 | 0.27 | 1.77 | 1.37 |
| F4 | 0.05 | 0.23 | 0.05 | 2.78 | 29.5 | 9.75 |
| F9 | 0.08 | 0.05 | 0.16 | 2.76 | 38.3 | 3.95 |

*A prefix, donor 1 vessel containing TMAO; B prefix, donor 1 vessel without TMAO (negative control); C prefix, donor 2 vessel containing TMAO; D prefix, donor 2 vessel without TMAO (negative control); E prefix, donor 3 vessel containing TMAO; F prefix, donor 3 vessel without TMAO (negative control).

**Supplementary Table 5.** Spearman correlation (corrected for ties) and adjusted *P* values (Benjamini–Hochberg) for FISH and metabolite data for batch culture samples

| Probe | **Spearman correlation values*** | | | | | |
| --- | --- | --- | --- | --- | --- | --- |
|  | **Acetate** | **DMA** | **Ethanol** | **Lactate** | **TMA** | **TMAO** |
| Eub338 | 0.37 | 0.14 | 0.38 | 0.15 | 0.06 | -0.38 |
| Bac303 | -0.18 | -0.30 | -0.17 | -0.21 | -0.36 | -0.16 |
| Ato291 | 0.78 | 0.33 | 0.79 | 0.60 | 0.57 | -0.46 |
| Bif164 | 0.93 | 0.37 | 0.87 | 0.81 | 0.72 | -0.48 |
| Ent | 0.82 | 0.59 | 0.65 | 0.75 | 0.81 | -0.09 |
| Bet42a | -0.69 | -0.22 | -0.62 | -0.78 | -0.62 | 0.24 |
| Prop853 | 0.40 | 0.01 | 0.47 | 0.40 | 0.24 | -0.32 |
| Lab158 | -0.05 | 0.14 | -0.21 | -0.43 | -0.08 | 0.02 |
| Fpra655 | 0.18 | 0.17 | 0.19 | -0.10 | 0.12 | -0.12 |
| Erec482 | 0.20 | 0.03 | 0.20 | -0.01 | 0.06 | -0.13 |
| Clit135 | -0.03 | 0.13 | 0.07 | -0.24 | -0.06 | 0.10 |
| Rbro730/Rfla729 | 0.15 | 0.04 | 0.24 | -0.07 | 0.05 | -0.22 |
| Chis150 | 0.85 | 0.56 | 0.74 | 0.76 | 0.85 | -0.13 |
| **Probe** | **Adjusted *P* values** | | | | | |
|  | **Acetate** | **DMA** | **Ethanol** | **Lactate** | **TMA** | **TMAO** |
| Eub338 | 2.51×10^-1^ | 7.05×10^-1^ | 2.51×10^-1^ | 7.05×10^-1^ | 8.05×10^-1^ | 2.51×10^-1^ |
| Bac303 | 5.23×10^-1^ | 5.23×10^-1^ | 5.23×10^-1^ | 5.23×10^-1^ | 5.23×10^-1^ | 5.23×10^-1^ |
| Ato291 | 3.88×10^-4^ | 1.84×10^-1^ | 3.88×10^-4^ | 1.67×10^-2^ | 1.99×10^-2^ | 6.89×10^-2^ |
| Bif164 | 1.13×10^-7^ | 1.33×10^-1^ | 8.41×10^-6^ | 8.01×10^-5^ | 1.04×10^-3^ | 5.55×10^-2^ |
| Ent | 1.63×10^-4^ | 1.26×10^-2^ | 5.32×10^-3^ | 6.66×10^-4^ | 1.63×10^-4^ | 7.15×10^-1^ |
| Bet42a | 5.06×10^-3^ | 3.71×10^-1^ | 9.87×10^-3^ | 7.82×10^-4^ | 9.87×10^-3^ | 3.71×10^-1^ |
| Prop853 | 2.09×10^-1^ | 9.64×10^-1^ | 2.09×10^-1^ | 2.09×10^-1^ | 4.15×10^-1^ | 3.02×10^-1^ |
| Lab158 | 9.35×10^-1^ | 9.35×10^-1^ | 9.35×10^-1^ | 4.38×10^-1^ | 9.35×10^-1^ | 9.35×10^-1^ |
| Fpra655 | 6.83×10^-1^ | 6.83×10^-1^ | 6.83×10^-1^ | 6.83×10^-1^ | 6.83×10^-1^ | 6.83×10^-1^ |
| Erec482 | 9.71×10^-1^ | 9.71×10^-1^ | 9.71×10^-1^ | 9.71×10^-1^ | 9.71×10^-1^ | 9.71×10^-1^ |
| Clit135 | 9.09×10^-1^ | 9.09×10^-1^ | 9.09×10^-1^ | 9.09×10^-1^ | 9.09×10^-1^ | 9.09×10^-1^ |
| Rbro730/Rfla729 | 8.65×10^-1^ | 8.65×10^-1^ | 8.65×10^-1^ | 8.65×10^-1^ | 8.65×10^-1^ | 8.65×10^-1^ |
| Chis150 | 2.80×10^-5^ | 2.00×10^-2^ | 6.10×10^-4^ | 5.37×10^-4^ | 2.80×10^-5^ | 6.21×10^-1^ |

*Green cells show statistically significant results.

**REFERENCES**

Daims, H., Brühl, A., Amann, R., Schleifer, K.-H. and Wagner, M. (1999) The domain-specific probe EUB338 is insufficient for the detection of all *Bacteria*: development and evaluation of a more comprehensive probe set. *Syst Appl Microbiol* 22, 434–444.

Franks, A.H., Harmsen, H.J.M., Raangs, G.C., Jansen, G.J., Schut, F. and Welling, G.W. (1998) Variations of bacterial populations in human feces measured by fluorescent in situ hybridization with group specific 16S rRNA-targeted oligonucleotide probes. *Appl Environ Microbiol* 64, 3336–3345.

Harmsen, H.J.M., Elfferich, P., Schut, F. and Welling, G.W. (1999) A 16S rRNA-targeted probe for detection of lactobacilli and enterococci in faecal samples by fluorescent *in situ* hybridization. *Microb Ecol Health Dis* 11, 3–12.

Harmsen, H.J.M., Raangs, G.C., He, T., Degener, J.E. and Welling, G.J. (2002) Extensive set of 16S rRNA-based probes for detection of bacteria in human feces. *Appl Environ Microbiol* 68, 2982–2990.

Harmsen, H.J.M., Wildeboer-Veloo, A.C.M., Grijpstra, J., Knol, J., Degener, J.E. and Welling, G.W. (2000) Development of 16S rRNA-based probes for the *Coriobacterium* group and the *Atopobium* cluster and their application for enumeration of *Coriobacteriaceae* in human feces from volunteers of different ages. *Appl Environ Microbiol* 66, 4523–4527.

Hold, G.L., Schwiertz, A., Aminov, R.I., Blaut, M. and Flint, H.J. (2003) Oligonucleotide probes that detect qualitatively significant groups of butyrate-producing bacteria in human feces. *Appl Environ Microbiol* 69, 4320–4324.

Hoyles, L. and McCartney, A.L. (2009) What do we mean when we refer to *Bacteroidetes* populations in the human gastrointestinal microbiota*? FEMS Microbiol Lett* 299, 175–183.

Kempf, V.A., Trebesius, K. and Autenrieth, I.B. (2000) Fluorescent in situ hybridization allows rapid identification of microorganisms in blood cultures. *J Clin Microbiol* 38, 830–838.

Langendijk, P.S., Schut, F., Jansen, G.J., Raangs, G.W., Kamphuis, G.R., Wilkinson, M.H.F. and Welling, G.W. (1995) Quantitative fluorescent in situ hybridisation of *Bifidobacterium* spp. with genus specific 16S rRNA targeted probes and its application in fecal samples. *Appl Environ Microbiol* 61, 3069–3075.

Loy, A., Maixner, F., Wagner, M. and Horn, M. (2007) probeBase – an online resource for rRNA-targeted oligonucleotide probes: new features 2007. *Nucleic Acids Res* 35, D800–D804.

Manz, W., Amann, R., Ludwig, W., Wagner, M. and Schleifer, K.-H. (1992) Phylogenetic oligodeoxynucleotide probes for the major subclasses of *Proteobacteria*: problems and solutions. *Syst Appl Microbiol* 15, 593–600.

Manz, W., Amann, R., Ludwig, W., Vancanneyt, M. and Schleifer, K.H. (1996) Application of a suite of 16S rRNA-specific oligonucleotide probes designed to investigate bacteria of the phylum cytophaga-flavobacter-bacteroides in the natural environment. *Microbiology* 142, 1097–1106.

Thorasin, T., Hoyles, L. and McCartney, A.L. (2015) Dynamics and diversity of the '*Atopobium* cluster' in the human faecal microbiota, and phenotypic characterization of '*Atopobium* cluster' isolates. *Microbiology* 161, 565–579.

Walker, A.W., Duncan, S.H., McWilliam Leitch, E.C., Child, M.W. and Flint, H.J. (2005) pH and peptide supply can radically alter bacterial populations and short-chain fatty acid ratios within microbial communities from the human colon. *Appl Environ Microbiol* 71, 3692–3700.
